# Is quality of colorectal cancer care good enough? Core measures development and its application for comparing hospitals in Taiwan

**DOI:** 10.1186/1472-6963-10-27

**Published:** 2010-01-27

**Authors:** Kuo-Piao Chung, Yun-Jau Chang, Mei-Shu Lai, Raymond Nien-Chen Kuo, Skye H Cheng, Li-Tzong Chen, Reiping Tang, Tsang-Wu Liu, Ming-Jium Shieh

**Affiliations:** 1 Graduate Institute of Health Care Organization Administration, College of Public Health, National Taiwan University, Taipei, Taiwan; 2 Center for Health Insurance Research, College of Public Health, National Taiwan University, Taipei, Taiwan; 3 Department of General Surgery, Zhong-Siao Branch, Taipei City Hospital, Taipei, Taiwan; 4 Institute of Preventive Medicine, College of Public Health, National Taiwan University, Taipei, Taiwan; 5 Department of Radiation Oncology, Koo Foundation Sun Yat-Sen Cancer Center, Taipei, Taiwan; 6 Department of Internal Medicine, National Chung-Kung University Hospital, Tainan, Taiwan; 7 Division of Colon and Rectal Surgery, Department of Surgery, Chang Gung Memorial Hospital, Linkou, Taiwan; 8 Division of Cancer Research, National Health Research Institutes, Taiwan; 9 Department of Gastroenterology, National Taiwan University Hospital, Taipei, Taiwan

## Abstract

**Background:**

Although performance measurement for assessing care quality is an emerging area, a system for measuring the quality of cancer care at the hospital level has not been well developed. The purpose of this study was to develop organization-based core measures for colorectal cancer patient care and apply these measures to compare hospital performance.

**Methods:**

The development of core measures for colorectal cancer has undergone three stages including a modified Delphi method. The study sample originated from 2004 data in the Taiwan Cancer Database, a national cancer data registry. Eighteen hospitals and 5585 newly diagnosed colorectal cancer patients were enrolled in this study. We used indicator-based and case-based approaches to examine adherences simultaneously.

**Results:**

The final core measure set included seventeen indicators (1 pre-treatment, 11 treatment-related and 5 monitoring-related). There were data available for ten indicators. Indicator-based adherence possesses more meaningful application than case-based adherence for hospital comparisons. Mean adherence was 85.8% (79.8% to 91%) for indicator-based and 82.8% (77.6% to 88.9%) for case-based approaches. Hospitals performed well (>90%) for five out of eleven indicators. Still, the performance across hospitals varied for many indicators. The best and poorest system performance was reflected in indicators T5-negative surgical margin (99.3%, 97.2% - 100.0%) and T7-lymph nodes harvest more than twelve(62.7%, 27.6% - 92.2%), both of which related to surgical specimens.

**Conclusions:**

In this nationwide study, quality of colorectal cancer care still shows room for improvement. These preliminary results indicate that core measures for cancer can be developed systematically and applied for internal quality improvement.

## Background

Colorectal cancer ranks as the third cause of cancer deaths in the United States and the fourth cause worldwide [1,2]. Thanks to improvements in diagnostic and surgical modalities as well as the advancement of chemotherapy and radiotherapy, many colorectal cancer patients do survive more than five years. The five-year relative survival rate rose from 51% two decades ago to 65% in recent years [2]. Still there are concerns about the quality of clinical practice since evidence has shown discernible variations for patients with similar conditions [3-5]. Evidence suggests that underuse and overuse of care may occur for patients with cancer [4]. Also, compared with the outcomes of patients in clinical trials, the outcomes of cancer patients in daily practice may be less favorable [6,7]. Research on the quality of care throughout at least the last decade has demonstrated that increases in the knowledge of treatments with proven efficacy do not translate directly to the optimal delivery of such treatments to patients [6,8].

Promoted by the Institute of Medicine, USA, the field of quality measurement has been growing rapidly along with accumulation of experience and knowledge in the cancer care quality area [9]. Core measures, introduced by the Joint Commission on Accreditation of Healthcare Organizations, use standardized sets of valid, reliable and evidence-based "core" measures that have been incorporated in quality measurement and performance improvement systems as well as the accreditation process for several diseases [10,11]. There are several groups who have previously developed measures for colorectal cancer [12]. Yet, the studies regarding measurement of cancer quality of care based on a national database are scarce and we know of no published studies related to core measures for cancer. Having a single set of measures for the entire country is the only way to set national benchmarks and compare countries that have national programs [13]. Our research team adopted the concept of core measures to assess the quality of care provided to patients with breast cancer, colorectal cancer, cervical cancer, lung cancer, oral cancer and hepatocellular carcinoma in Taiwan.

Health insurance in Taiwan is provided within a single payer system that has covered over 96% of the population since 1995 [14]. Following the Cancer Prevention and Management Law, a revised cancer registry, the Taiwan Cancer Database (TCDB) was created in 2003 [15]. The Bureau of Health Promotion (BHP) in the Department of Health financially supported 27 hospitals (initially 17 major hospitals) to join the TCDB program with the prerequisite that each hospital is required to provide cancer prevention, cancer screening, diagnosis and treatment of cancer, education and clinical audits, and cancer research as well as a quality assessment system [16]. The TCDB is a nationwide oncology outcomes database that currently covers approximately 60% of six new invasive cancer diagnoses in Taiwan each year. Like its counterpart, the National Cancer Data Base (NCDB) in the USA, the TCDB is a large, powerful database providing multiple opportunities for clinical studies and can be used to benchmark hospitals on performance measures--an impetus for quality improvement initiatives at the hospital level [17]. Each hospital that voluntarily participates must submit data twice a year for six types of cancers that mentioned above. The prevalence of each disease is increasing, motivating public interest in cancer care quality nationwide. The core cancer measures were developed in parallel with the TCDB program through a government grant as a national initiative for cancer care quality improvement [15]. Although performance measurement for assessing care quality is booming, a system for measuring the quality of cancer care at the hospital level has not been well developed. The purpose of this study was to develop organization-based core measures for colorectal cancer patient care and apply these measures to compare hospital performance.

## Methods

### Patients and hospitals

The patients and hospitals registered in the TCDB program in Taiwan during 2004 were eligible for this study. Participation in the program is voluntary for each hospital but the number is increasing each year partly due to financial incentives. Some information is required in order to link each cancer registry record to insurance claims data. Due to problems with the timeliness and quality of data for nine of the hospitals, eighteen hospitals were selected as the main sources of data for this study (see Appendix).

### Core measures development

**Figure 1:**
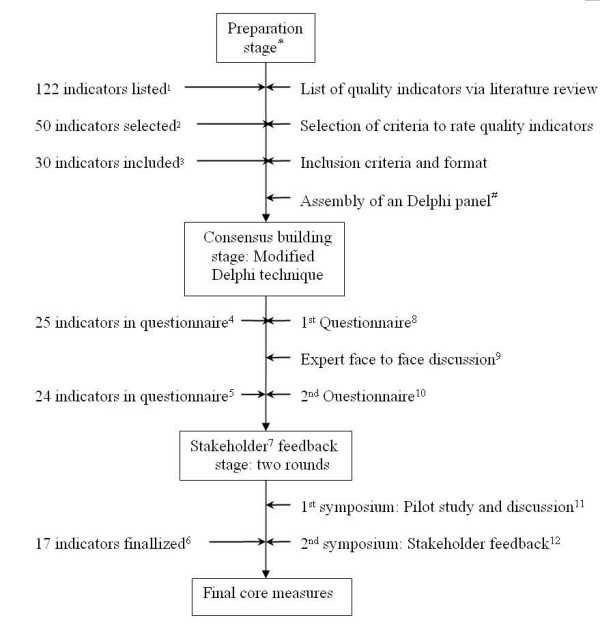
**Design and development of core measure for colorectal cancer**. *members of research team included KPC, SHC, LTC, RT, TWL, MJS and a scholar, ^#^twenty expert were invited (research team member excluded), see also the expert panel in Appendix, ^1 ^including 37 structure indicators, 78 process indicators, and 7 outcome indicators, ^2 ^including 9 structure indicators, 37 process indicators, and 4 outcome indicators, ^3^including 4 pre-treatment indicators, 21 treatment indicators, and 5 monitor indicators, ^4^including 3 pre-treatment indicators, 16 treatment indicators, and 6 monitor indicators, ^5^including 2 pre-treatment indicators, 16 treatment indicators, and 6 monitor indicators, ^6^including 1 pre-treatment indicator, 11 treatment indicators, and 5 monitor indicators, ^7^hospitals and clinical professionals are major stakeholders, ^8^questionnaires were mailed on August 16, 2004, response rate 95% (19/20), ^9 ^expert meeting was held in Taipei, September 22, 2004, ^10 ^questionnaires were mailed on October 26, 2004, response rate 85% (17/20), ^11 ^1^st ^symposium was held in Taipei, December 27, 2005, ^12 ^2^nd ^symposium was held in Taipei, March 27, 2006.

The process of core measures development has gone through a preparation stage, a consensus building stage, and two rounds of stakeholder feedback (see Figure 1), similar with our pilot study of breast cancer [16]. This study was sponsored by a grant (DOH93-HP-1057) and approved by Review Board of Data Release (equivalent to Ethics Committee) from the Bureau of Health Promotion, Department of Health, Taiwan. Seven university faculty members (including Author KPC, SHC, LTC, RT, TWL, MJS and a scholar) voluntarily constituted the research team, which held four meetings in the preparation stage. The research team then collaborated with experts and other stakeholders to develop rigorous, evidence-based, scientifically sound measures which could be used as the standards of cancer care quality nationwide. The search terms used included the Medical Subject Heading terms 'outcome and process assessment health care', 'quality indicator health care', 'quality of health care', 'practice guidelines', 'evidence-based medicine', 'colorectal neoplasms' and the keyword 'performance measures' from January 1985 to January 2002. All possible quality-related cancer indicators were summarized (including RAND (Research and Development Corporation, USA) and National Health Service guidelines) [18-30] and reduced to a final measure set via a modified Delphi method and stakeholder feedback. In addition to validity and feasibility, indicator selection was guided by other criteria such as scientific acceptability, importance and necessity, and usability [16].

The Delphi group, nominated by the Taiwan Clinical Oncology Group from the National Health Research Institute as well as related specialty societies, encompassed twenty clinical professionals who represented the organization of their specialty. Included were four gastro-intestinal physicians, three oncological physicians, three radiological oncologists, seven colorectal surgeons and three pathologists (see Appendix). The panel members met face-to-face during 2004. Indicators were anonymously rated on a 9-point scale (with 1 denoting not valid and 9 very valid) and only indicators with a median score of 7 or higher were selected as final candidate indicators. After initial analysis of the panel data, the expert panel and stakeholders from different hospitals met on March 27, 2006 to review and streamline the indicators.

### Comparisons across hospitals

Currently, the emphasis on quality measurement in clinical, including cancer, care is on evaluating adherence to "evidence-based" process measures. This quantitative approach to quality improvement moves the field forward by ranking quality measures according to both quantitative assessment of under-performance across institutions and the measures' potential impact on outcomes [31]. Reeves et al. recently summarized five methods for computing a composite quality score including "Indicator Average" and "Patient Average" [32], both of which we used in this study to derive adherence across hospitals and were designated as indicator-based and case-based adherence. Indicator-based adherence was derived from "all the care received in a given hospital for a specific indicator" divided by "all the care recommended in a given hospital for that indicator". Case-based adherence was computed as the percentage of indicators that were successfully met for each patient. These percentages were then averaged across all patients in a given hospital. To compare hospitals, three different scenarios were adopted. In the first scenario, each hospital was awarded one point for each indicator in which adherence to criteria was greater than the 50^th ^percentile. These points were then added to form a hospital score. Similar procedures were followed for Scenarios 2 and 3 except the thresholds were set at the 75^th ^and 90^th ^percentiles respectively. This approach made hospital comparisons an easier task.

## Results

### Patient and disease characteristics

A total of 5585 patients who had newly diagnosed colorectal cancer and complete information regarding their treatment during 2004 entered into this analysis. The demographic characteristics are listed as Table 1. Mean age of the recruited sample was 64 years old. Males were more prone to have colorectal cancer (male to female: 58.3% to 41.7%), even in each stage. More than sixty percent of the patients were older than sixty years old. Patients younger than 40 years old comprised only 6% of the study group. By disease stage, Stage III was the most common stage at the time colorectal cancer was discovered. It constituted 29.9% of the sample, followed by Stage II (24.9%), Stage IV (20.6%) and Stage I (14.1%). Carcinoma in situ was only found in about 3% of the newly diagnosed patients (Table 1).

**Table 1:** Characteristics of colorectal cancer patients undergoing surgery in 2004 by cancer staging.

	Gender		Age (years)					
	
	Male	Female	<30	31-40	41-50	51-60	>60	Total
stage 0	97	60	2	7	22	38	88	78(1.4%)*
stage 1	463	324	6	27	65	167	522	787(14.1%)*
stage 2	811	580	15	49	142	229	956	1,391(24.9%)*
stage 3	960	711	14	94	200	340	1023	1,671(29.9%)*
stage 4	633	488	31	61	164	219	676	1,151(20.6%)*
unspecified stage	263	165	10	19	44	75	280	428(7.7%)*

	3257 (58.3%)^#^	2328 (41.7%)^#^	157 (2.8%)^#^	257 (4.6%)^#^	637 (11.4%)^#^	1068 (19.1%)^#^	3545 (63.5%)^#^	5585 (100%)

* the number in the parenthesis indicates the percentage of each stage (e.g. 1.4%= 78/5585);

^#^the number in the parenthesis indicates the percentage of gender or age level (e.g. 58.3%= 3257/5585)

### Core measures

The final set of core measures for colorectal cancer encompassed seventeen indicators belonging to three groups: one for "Pre-treatment", eleven for "Treatment", and five for "Monitoring". Table 2 (see Additional file 1 for definition) displays the description, rationale, numerator, denominator, and evidence level (or grade of recommendation) for each indicator. However, not all core measures of colorectal cancer developed in this fashion were included in this study. All five indicators for "Monitoring" were excluded due to the constraint of short study interval. Two indicators (T2 and T8) requiring intensive chart reviews were not also included. Finally, ten indicators (T3 was divided into T3a, T3b sub-indicator) could be constructed from attainable data for colorectal cancer. Nearly all data required were retrieved from the TCDB with the exception of the T1 indicator, in which partial information was obtained from claim data.

**Table 2:** Core measure indicators for colorectal cancer

Indicator	Description	Numerator	Denominator	Evidence level or grade of recommendation	Rationale
PT1	Proportion of colorectal patients who have pre-operative chest X-ray and abdominal ultrasound, CT scan or MRI^18^	Number of colorectal patients who have pre-operative chest x-ray and abdominal ultrasound, CT scan or MRI (including off-site)	Number of all colorectal patients	Grade C	Comprehensive-ness of pre-operative evaluation

T1	Proportion of colorectal patients who have undergone surgical resection for colon or rectal cancer have documentation that colonoscopy or barium enema with sigmoidoscopy was offered within 6 months before or after surgery^19,20^	Number of colorectal patients who have undergone surgical resection for colon or rectal cancer have documentation that colonoscopy or barium enema with sigmoidoscopy (including off-site) was offered within 6 months before or after surgery	Number of colorectal patients who have undergone surgical resection for colon or rectal cancer	Level II-2	Synchronous colon or rectal cancer

T2*	Proportion of colorectal patients who have undergone wide surgical resection for malignant polyp of colon or rectum within 6 weeks of pathological report (polypectomy) revealing incomplete or positive margin or venous or lymphatic invasion or poorly undifferentiated^19^	Proportion of colorectal patients who have undergone wide surgical resection for malignant polyp of colon or rectum within 6 weeks of pathological report (polypectomy) revealing incomplete or positive margin or venous or lymphatic invasion or poorly undifferentiated	Number of colorectal patients who pathological report for malignant polyp of colon or rectum revealing incomplete or positive margin or venous or lymphatic invasion or poorly undifferentiated	Level II-2, III	Increase curability

T3a	Proportion of patients who are diagnosed with colon cancer and do not have metastatic disease were offered a curative resection within 6 weeks of diagnosis. (For patient with diagnosis and treatment of care in the same hospital)^19,23^	Number of patients who are diagnosed with colon cancer and do not have metastatic disease were offered a curative resection within 6 weeks of diagnosis. (For patient with diagnosis and treatment of care in the same hospital)	Number of patients who are diagnosed with colon cancer and do not have metastatic disease. (For patient with diagnosis and treatment of care in the same hospital)	Level II-2, III	Improve survival

T3b	Proportion of patients who are diagnosed with colon cancer and do not have metastatic disease were offered a curative resection within 6 weeks of diagnosis^19,23^	Number of patients who are diagnosed with colon cancer and do not have metastatic disease were offered a curative resection within 6 weeks of diagnosis	Number of patients who are diagnosed with colon cancer and do not have metastatic disease	Level II-2, III	Improve Survival

T4	Proportion of stage I to III colorectal patients who have histopathology reports which give the degree of involvement of surgical margins, including circumferential margins, the number of lymph nodes examined and the number involved^21^, ^22^	Number of stage I to III colorectal patients who have histopathology reports which give the degree of involvement of surgical margins, including circumferential margins, the number of lymph nodes examined and the number involved	Number of stage I to III colorectal patients excluding patients undergo polypectomy	Grade B	Provide information for subsequent intervention, which related to tumor recurrence

T5	Proportion of stage I to III colorectal patients who undergo a wide surgical resection that have documented to be "negative margins"^19,23^	Number of stage I to III colorectal patients who undergo a wide surgical resection that have documented to be "negative margins"	Number of stage I to III colorectal patients excluding no pathological report regarding status of margin	Level II-2, III	Radical excision

T6	Proportion of colorectal patients who undergo a surgery that have a pathology report with the information on tumor size and node differentiation^22,24^	Number of colorectal patients who undergo a surgery that have a pathology report with the information on tumor size and node differentiation	Number of colorectal patients who undergo a surgery	Class B	Provide information for subsequent intervention and follow-up

T7	Proportion of I to III stage colorectal cancer patients with twelve or more lymph nodes on pathology report^25^	Number of I to III stage colorectal cancer patients with twelve or more lymph nodes on pathology report	Number of I to III stage colorectal cancer patients excluding polypectomy, or neo-radiotherapy	Level III or IV	Radical excision and pathological accountability

T8*	Proportion of pathological report according to CAP checklist or similar one^21,26^	Number of pathological report according to CAP checklist or similar one	Number of colorectal patients		Pathological report comprehensiveness

T9	Proportion of stage III colon cancer patients who was offered chemotherapy within 6 weeks after surgery^19,27,28^	Number of stage III colon cancer patients who was offered chemotherapy within 6 weeks after surgery	Number of stage III colon cancer patients who was offered chemotherapy after surgery (in the same hospital)	Level I, II-2, III	Increase Survival

T10	Proportion of patients who are diagnosed with Stage II or III rectal cancer, was offered treatment including surgery, radiotherapy, or CCRT treatment within 6 weeks of diagnosis^19,24^	Number of patients who are diagnosed with Stage II or III rectal cancer, was offered treatment including surgery, radiotherapy, or CCRT treatment within 6 weeks of diagnosis	Number of patients who are diagnosed with Stage II or III rectal cancer, was offered treatment in the same hospital	Level II-2, III	Avoid treatment delay

T11	Proportion of patients who are diagnosed with rectal cancer that appears clinically to be Stage II or III, and was offered surgical resections within 16 weeks after beginning of CCRT^19,23^	Number of patients who are diagnosed with rectal cancer that appears clinically to be Stage II or III, and was offered surgical resections within 16 weeks after beginning of CCRT	Number of patients who are diagnosed with rectal cancer that appears clinically to be Stage II or III, and was offered CCRT before surgery	Level II-2, III	Provide treatment for cure intent

F1*	Proportion of patients who had completed all courses of therapy for Stage I to III lesions had medical check-up again within 6 months of completion^19,29^	Number of patients who had completed all courses of therapy for Stage I to III lesions had medical check-up again within 6 months of completion	Number of patients who had completed all courses of therapy for Stage I to III lesions	Level II-2, III	Decrease mortality

F2*	Proportion of patients who had completed surgery for Stage I to III lesions had medical check-up again including colonoscopy or LGI series within 2 years period and subsequent 3 years interval^19^	Number of patients who had completed surgery for Stage I to III lesions had medical check-up again including colonoscopy or LGI series within 2 years period and subsequent 3 years interval	Number of patients who had Stage I to III lesions	Level II-2, III	Decrease recurrence

F3*	Proportion of patients who are treated by polypectomy procedure for malignant polyp was offered colonoscopy within 12 months of procedure^19,30^	Number of patients who are treated by polypectomy procedure for malignant polyp was offered colonoscopy within 12 months of procedure	Number of patients who are treated by polypectomy procedure for malignant polyp	Level III	Decrease recurrence

F4*	Five-year over-all survival rate, stage specific (Stage I to IV)^28^	Number of patients who survive at five-year period, stage specific (Stage I to IV)	Number of patients, stage specific (Stage I to IV)	Class C	Assess survival

F5*	Five-year local recurrence rate, stage specific (Stage I to III)^18^	Number of patients who had recurrence at five-year period, stage specific (Stage I to III)	Number of patients, stage specific (Stage I to III)	Grade B	Assess recurrence

*: indicators excluded in the results due to data un-available.

1. Stage I to IV are based on classification of TNM system, AJCC (the American Joint Committee on Cancer);

2. Level I, Level II-2, Level III and Level IV: adopted from RAND^19^, Desch^29^; Class B: adopted from SIGN 2003^22^; Class C: adopted from Guideline 2001^28^; Grade B and Grade C: adopted from RCSI 2002^18^; see Additional file 1 for definition.

3. PT: pre-treatment; T: treatment; F: follow-up; CT: computed tomography; MRI: magnetic resonance imaging; CAP: College of American pathology; CCRT: concurrent chemo-irradiation; LGI series: Lower gastrointestinal series.

### Hospital performances and comparisons

**Figure 2:**
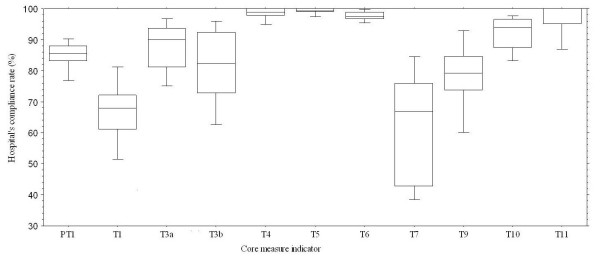
**Box-plot illustrates hospital's adherence for each core measure indicator (indicator-based) of colorectal cancer**. Mean of five indicators (T4, T5, T6, T10, T11) had adherence greater than 90%. PT: pre-treatment, T: treatment.

Hospital performances were displayed as indicator-based and case-based adherence. To show system performance for colorectal cancer, the box plots in Figure 2 illustrate the range of hospital performance with respect to indicator-based adherence. From this informative plot, we learned how good or how bad our hospitals performed with respect to each indicator--the display helps make explicit which indicators might be improved. Five indicators (45% or 5/11) in this plot had mean adherence greater than 90% (T4: 98.3%, T5:99.3%, T6: 97.6%, T10: 91.8%, T11: 96.8%). Still, there were many indicators showing a wide range of performance. We also note that two indicators had mean adherence less than 70% (T1 66.6%, 36.6% to 85.0%; T7: 62.7%, 27.6% to 92.2%). The best and poorest system performance occurred for T5-negative surgical margin (99.3%, ranged from 97.2% to 100.0%) and T7-lymph nodes harvest more than twelve (62.7%, ranged from 27.6% to 92.2%). Both indicators relate to surgical specimens.

Table 3 summarizes the details of adherence to the core measures and hospital scores for inter-hospital comparisons. Data were presented as means, 95 percent confidence intervals and ranges as indicated. Then we displayed results in a table of inter-hospital comparisons. Adherence to indicator-based and case-based approaches for each hospital ranged from 79.8% to 91.0% and 77.6% to 88.9%, respectively. Results of indicator-based approach are always larger than case-based approach except for one hospital (H06). On average, patients received 85.8% of recommended care for indicator-based adherence, and 82.8% for case-based adherence. The three different scenarios of hospital scoring methods could only be applied to indicator-based adherence. The average hospital scores were 5.3 (2 to 8), 3.5 (0 to 6), and 2.2 (0 to 4) with respect to the 50^th^, 75^th^, and 90^th ^percentiles respectively. Hospital rankings were not consistent among the three methods. Adoption of alternative methods of aggregating scores can lead to different interpretations.

**Table 3:** Results of organizational performance of colorectal cancer care by core measure indicators

Hospitals\Performance	Indicator-based					Case-based	
		
	Adherence^1^*		Hospital score^1#^	Hospital score^2#^	Hospital score^3#^	Adherence^2^*	
	%	(95 CI)				%	(95 CI)
H01^§^	86.5	(79.4-93.7)	5	2	2	83.5	(80.4-86.7)
H02	87.1	(79.3-95.0)	5	5	2	84.2	(82.4-86.0)
H03	82.3	(68.9-95.8)	6	4	2	79.8	(76.7-82.9)
H04	82.4	(70.3-94.6)	5	4	4	79.7	(77.2-82.1)
H05	88.8	(82.2-95.4)	7	3	1	86.1	(82.7-89.5)
H06	85.7	(76.3-95.1)	6	4	4	88.1	(85.3-90.9)
H07	90.2	(83.0-97.4)	8	6	4	85.6	(82.3-89.0)
H08	91.0	(83.2-98.7)	8	6	3	88.9	(87.8-90.1)
H09	85.9	(77.5-94.3)	4	3	1	81.2	(78.5-83.9)
H10	86.1	(78.2-94.0)	6	4	4	84.7	(82.6-86.9)
H11	79.8	(67.7-91.9)	2	0	0	79.4	(77.5-81.3)
H12	84.7	(75.0-94.5)	4	1	1	82.2	(79.7-85.3)
H13	88.9	(82.1-95.7)	6	4	2	84.1	(80.4-87.7)
H14	85.3	(76.3-94.2)	6	6	3	85.4	(82.6-88.1)
H15	82.4	(74.1-90.7)	3	0	0	80.7	(78.4-83.1)
H16	81.8	(71.3-92.4)	4	4	2	77.6	(73.0-82.1)
H17	85.0	(73.8-96.1)	6	3	2	78.7	(75.1-82.3)
H18	86.6	(76.9-96.3)	5	4	3	81.6	(79.6-83.7)

Mean	85.8	(78.3-93.3)	5.3	3.5	2.2	82.8	

*: Adherence^1^(Indicator-based adherence); Adherence^2 ^(case-based adherence); 95 CI (95% confidence interval) listed in the parenthesis

^#^: Hospital score are composite score for indicator-based level, defined as following: one point was awarded if adherence >50^th ^percentile (Hospital score^1^) or >75^th ^percentile (Hospital score^2^) or > 90^th ^percentile (Hospital score^3^) for each indicator, and then sum up.

^§^: H01 to H18 denote the corresponding hospital (H). We report the results by designation to maintain the confidentiality of the hospitals and providers who participated in TCDB.

## Discussion

The core measures for colorectal cancer discussed in this study are one of the six cancer-specific sets of indicators developed in a government-sponsored program with scoring technique improvements, larger expert participation. Refinement of core measures development from our prior study of breast cancer included: 1. expand the number of Delphi panelists from seven to twenty; 2. adopt the RAND Appropriateness technique (scale 1 to 9) to select indicators from previously developed technique (scale 1 to 5, median equal or greater than four and 86% agreement threshold) [5]; 3. change threshold for selecting indicators to a score of seven or higher. More stakeholders' participation and discussion during stakeholder feedback symposium suggest enthusiasm and the commitment of healthcare professionals in the development of methods for quality improvement in colorectal cancer care. However, the level of evidence for indicators could not match those of the pilot study we developed previously for breast cancer, which included six level I, four level II and five level III recommendations. Nevertheless, it is our belief that these measures represent a valuable contribution to the growing library of clinical cancer measures which can be used for public awareness, potential improvement and accreditation standards for cancer care centers or specialty groups.

Any new performance measure set, including the current cancer core measure sets, frequently encounter a major hurdle, i.e. data resources. It would be easier if the information needed to construct measures came from ICD-9 codes of a billing system. But actually for cancer care research, requirements include the stage, tumor size, node numbers, and status of section margins, none of which are captured in claim-based systems. Although these data are available in medical records, such detailed review of charts on a national scale would be cumbersome and expensive [33]. The TCDB program solved this dilemma; its establishment was the result of the increasing demands for research that would help inform reporting and pay for performance systems. Breast cancer was the first disease entity undertaken by the TCDB program. The number of newly diagnosed breast cancers represented 81.4% of all new cases in 2004, partly due to greater efforts in reporting. Reports of colorectal cancer didn't catch up this pace, but finally we identified 5585 patients newly diagnosed in 2004 within the TCDB. This sample accounted for 58.6% (5585/9535) of all newly diagnosed patients with cancer that occurred in Taiwan that year. We consider this sample representative of the population who has been and is being treated for colorectal malignancy in this country. The scope of the TCDB program will hopefully extend to all types of cancer in the future and will continue to provide feedback to participating hospitals.

Our results suggest that patients with colorectal cancer receive 86% (indicator-based adherence) and 83% (case-based adherence) of recommended care. Adherence to four indicators approached 100%, indicating that excellent quality of care is accomplishable. Yet, we observed four indicators with less than 85% adherence, and for these indicators, there was substantial variability in quality across hospitals. The adherence to twelve core measures for breast cancer in our prior report was inferior (65.8%, indicator-based).

Our results can be compared with a growing literature that describes the quality of cancer care, as well as studies that describe care quality of chronic conditions. For example, results of the National Initiative for Cancer Care Quality (NICCQ) showed that patients with breast cancer receive 86% of recommended care, whereas patients with colorectal cancer receive 78% of recommended care [3]. Using a methodology similar to that of NICCQ, two national studies suggest that the quality of care for cancer may be similar or better than that observed for other chronic medical conditions. Jencks et al. reported 73% of Medicare beneficiaries received health care services specified by one of 24 quality measures addressing heart disease, stroke, and pneumonia [34]. Another study by McGlynn et al. concluded that participants in 12 metropolitan areas of the USA received approximately 55% of recommended care for thirty acute and chronic conditions according to the results from a nation-wide telephone survey [5]. But in that study, they reported 53.9% (95% CI, 45.7-60.4) adherence to twelve quality indicators for colorectal cancer.

Indicator-based adherence is another issue that must be discussed. Since the quality measurement of cancer care is emerging, few studies have reported results of hospital adherence for each indicator. Adherence measured by four indicators in our results (T1-diagnostic evaluation, T3b-surgery, T7-pathological reporting, T9-adjuvant therapy) were less than 85%. In contrast, all diagnostic evaluation, adjuvant therapy (but not surgery), and pathological reporting in the NICCQ showed adherence less than 85% [3]. Another unexpected finding is the adherence to indicator T9, the only indicator with Level I evidence support, was less than 85% (77.4%). Of note, we explored the best (T5) and poorest (T7) adherence for system performance, which might suggest that surgeons did well to achieve safety margins for cancer specimens but failed to remove more than twelve lymph nodes as recommended. But actually there are three major influences on the total number of nodes harvested: the surgeon, with regards to technique and philosophy; the pathologist, with regard to specimen evaluation technique (fat clearing or cherry-picking) and philosophy; and patient characteristics, with regard to tumor site, T stage, immune response, and age [35]. A study by Bilimoria et al. lamented that more than 60% of US institutions failed to achieve a compliance benchmark for the 12-node measure and called for efforts aimed at improvement in colon cancer nodal evaluation [36]. According to their benchmark (examination of ≥12 nodes in ≥75% of patients), we had as much as 72% (13/18) of institutions that failed such a benchmark and actually this percentage might be under-estimated for the daily practice for all cancer care in Taiwan. Improvement should be encouraged for these controllable factors.

Two different types (indicator-based and case-based) of adherence have been presented in this organizational level study. Results of case-based adherence and indicator-based adherence were similar (usually slightly smaller for case-based). But it seems there may be more practical applications for indicator-based adherence since as shown in Table 3, indicator-based adherence can provide more details in terms of potential improvements to be considered than case-based adherence. The box-plot in Figure 2 was also created according to the indicator-based adherence methodology. For quality comparisons at the organizational level, we would suggest indicator-based adherence measures, which provide more needed information on quality improvement than case-based adherence.

Individual quality measures have significant limitations for assessing performance. Despite growing interest in composite measures, methods for combining multiple domains of surgical quality are not well established [37]. Composite scores are an aggregation of underlying performance indicators into a single index and have been used widely in the public sector to create one more easily understood number or rating [38,39]. But in combining quality indicators to rank hospitals, how one aggregates quality measures for easy understanding is still disputable. Following the criterion-referenced method, we arbitrarily used three different thresholds to rank performance of participating hospitals. Composites are bedeviled by questions of weighting [30]. All weighting systems are arbitrary, and equal weighting is transparent even if it is indefensible at the margins [40]. In the current study, every opportunity to deliver recommended care is weighted equally. However, with equal weighting, quality measured by a composite could seem high even if rare but critical processes were lethally unsafe. Given the increasing use of composites, research is needed to define which indicators can legitimately be made into composites, to develop and test clear rationales for weighting schemes, and to build understanding of how changing weights alters conclusions about performance [41].

As quality measures are gaining momentum in the healthcare industry, our findings have important implications for health policy. The core measures can be used for internal improvement of cancer care in the near future. Also, core measures may provide accountability for the basis of pay-for-performance systems and public reporting, as well as for accreditation.

Several limitations of this study should be discussed. First, the database of this study was secondary data. Although the personnel of the cancer registry for each organization have undergone accreditation and continuous education, we cannot overlook the possibilities of mistyping or underreporting. Second, only hospitals participating in the project reported to the TCDB. These hospitals may exhibit a higher level of specialization than hospitals that are not in the project. If we combined reporting and non-reporting hospitals, the differences in adherence would likely be increased. Third, we focused more on the effectiveness aspect of quality, while other aims for improvement such as safety and patient-centered care were not considered simultaneously [42]. Finally, as we mentioned earlier, the evidence level for colorectal cancer doesn't parallel that available for breast cancer which limits the study's generalizability. Research studies such as randomized clinical trials of cancer care should be encouraged in the future to enhance the evidence level of research on colorectal cancer care.

## Conclusions

Standardized performance measurement is an emerging methodology for comparing health care quality among different facilities. To the best of our knowledge, this is the first study on colorectal cancer care quality based on core measures with a nationwide database. Developing core measures for cancer care is the first step to achieving standardized measures for external monitoring, as well as for providing feedback and serving as benchmarks for cancer care quality improvement. By applying a core measures approach more comprehensively in this fashion, hopefully we can expect to standardize clinicians' daily practice as well as reduce care disparities and improve outcomes for the general population. Future research may capture opportunities to improve cancer care quality by including perspectives from patients, physicians and systems as well as the relationships between core measures and outcomes.

## Abbreviations

NCDB: National Cancer Data Base; TCDB: Taiwan Cancer Database; RAND: Research and Development.

## Competing interests

The authors declare that they have no competing interests.

## Authors' contributions

KP, MS conceived and designed the Study. KP was the principal investigator and wrote the manuscript. YJ & RNC partook in the study design and collected data. SH, LT, R, TW and MJ participated the research team. All Authors read and approved the final manuscript.

## Appendix

1. Expert panel members and affiliations:

• MD, PhD, Professor, Department of Medicine, National Taiwan University Hospital;

• MD, PhD, Associate Professor, Department of Surgery, National Taiwan University Hospital;

• MD, PhD, Department of Radiation Oncology, National Taiwan University Hospital;

• MD, PhD, Professor, Department of Colorectal Surgery, Taipei Veteran General Hospital;

• MD, Department of Radiation Oncology, Taipei Veteran General Hospital;

• MD, Director, Department of Colorectal Surgery, Taichung Veteran General Hospital;

• MD, Professor, Director, Department of General Surgery, Tri- Service General Hospital;

• MD, Associate Professor, Director, Department of Radiology, Tri- Service General Hospital;

• MD, Director, Department of Oncology, Taipei Mackay Memorial Hospital;

• MD, PhD, Associate Professor, Director, Department of Oncology, Far Eastern Memorial Hospital;

• MD, Director, Department of Internal Medicine, Taipei Cathy General Hospital;

• MD, Director, Department of Pathology, Taipei Cathy General Hospital;

• MD, PhD, Department of oncology, Chung-Gung Memorial Hospital;

• MD, PhD, Professor, Director, Department of Colorectal Surgery, Chung-Gung Memorial Hospital;

• MD, PhD, Professor, Director, Department of Medicine, Chung-Gung Memorial Hospital;

• MD, PhD, Associate Professor, Director, Department of Oncology, Chung-Gung Memorial Hospital;

• MD, PhD, Professor, Director, Department of Surgery, National Chen-Gung University Hospital;

• MD, PhD, Professor, Director, Department of Pathology, National Chen-Gung University Hospital;

• MD, Director, Department of Internal Medicine, Kaohsiung Medical University Hospital;

• MD, PhD, Director, Department of Oncology, Chung-Gung Memorial Hospital.

2. Hospital enrolled in the context including:

• National Taiwan University Hospital;

• National Chen-Gung University Hospital;

• Taipei Veteran General Hospital;

• Taichung Veteran General Hospital;

• Kaohsiung Veteran General Hospital;

• Taipei Mackay Memorial Hospital;

• Tri- Service General Hospital;

• Koo Foundation Sun Yet-Sen Cancer Center;

• Buddhist Tzu Chi General Hospital, Da-Lin Branch;

• Taipei Cathy General Hospital;

• Shin Kong Wu Ho-Su Memorial Hospital;

• Chung-Gung Memorial Hospital;

• Chung-Gung Memorial Hospital, Kaohsiung Branch;

• China Medical University Hospital, Taiwan;

• Changhua Christian Hospital;

• Chia-Yi Christian Hospital;

• Chi Mei Medical Center;

• Kaohsiung Medical University Hospital.

## Pre-publication history

The pre-publication history for this paper can be accessed here:

http://www.biomedcentral.com/1472-6963/10/27/prepub

## Supplementary Material

Additional file 1 **Levels of evidence or grades of recommendations**. This additional file 1 showed what specifications of evidence levels or recommendation grades we used during development of core measures. Click here for file
