# Supplementary material for: Is quality of colorectal cancer care good enough? Core measures development and its application for comparing hospitals in Taiwan
Source: BMC Health Serv Res. 2010 Jan 27;10:27. doi: 10.1186/1472-6963-10-27 (PMC2835701; doi:10.1186/1472-6963-10-27)
Supplement: Additional file 1 — Levels of evidence or grades of recommendations. This additional file 1 showed what specifications of evidence levels or recommendation grades we used during development of core measures. [file 1472-6963-10-27-S1.DOC]

| Guidelines 2001^28^  RCSI 2002^18^ | SIGN 2003^22^ | RAND^19^ | Desch^29^ | Description |
| --- | --- | --- | --- | --- |
| A | A | I Randomized  controlled trials | I | Evidence obtained from meta-analysis of multiple, well-designed, controlled studies.  Randomized trials with low false-positive and low false-negative errors (high power) |
|  |  |  | II | Evidence obtained from at least one well-designed experimental study. Randomized trials with high false-positive and/or false-negative errors (low power) |
| B | B | II-1  Nonrandomized  controlled  trial | III | Evidence obtained from well-designed, quasi- experimental studies such as non- randomized, controlled, single-group, pre-post, cohort, time, or matched case-control  series |
|  |  | II-2 Cohort or  case analysis |  |  |
|  | C | III Multiple time  series | IV | Evidence from well-designed, non-experimental  studies such as comparative and correlational descriptive and case studies |
| C | D | IV Opinions or  descriptive | V | Evidence from case reports and clinical  examples |

Levels of evidence or grades of recommendations
